# Synergy between repellents and non-pyrethroid insecticides strongly extends the efficacy of treated nets against *Anopheles gambiae*

**DOI:** 10.1186/1475-2875-6-38

**Published:** 2007-03-29

**Authors:** Cédric Pennetier, Vincent Corbel, Pélagie Boko, Abibatou Odjo, Raphaël N'Guessan, Bruno Lapied, Jean-Marc Hougard

**Affiliations:** 1 Institut de Recherche pour le Développement (IRD), Cotonou, Bénin; 2 Centre de Recherches Entomologiques de Cotonou (CREC), Cotonou, Bénin; 3 Laboratoire de Lutte contre les Insectes Nuisibles (LIN), Institut de Recherche pour le Développement (IRD), Montpellier, France; 4 London School of Hygiene and Tropical Medicine, London, UK; 5 RCIM, UPRES EA 2647, Université d'Angers, F-49045 Angers cedex, France

## Abstract

**Background:**

To manage the *kdr *pyrethroid-resistance in Anopheline malaria vectors, new compounds or new strategies are urgently needed. Recently, mixing repellents (DEET) and a non-pyrethroid insecticide (propoxur) was shown to be as effective as deltamethrin, a standard pyrethroid, under laboratory conditions, because of a strong synergy between the two compounds. In the present study, the interactions between two repellents (DEET and KBR 3023) and a non-pyrethroid insecticide (pyrimiphos methyl or PM) on netting were investigated. The residual efficacy and the inhibition of blood feeding conferred by these mixtures were assessed against *Anopheles gambiae *mosquitoes.

**Methods:**

DEET and KBR 3023 were mixed with pyrimiphos methyl (PM), a organophosphate (OP) insecticide. The performance of mono- and bi-impregnated nets against adult mosquitoes was assessed using a miniaturized, experimental hut system (laboratory tunnel tests) that allows expression of behavioural responses to insecticide, particularly the mortality and blood feeding effects.

**Results:**

Both mixtures (PM+DEET and PM+KBR3023) induced 95% mortality for more than two months compared with less than one week for each compound used alone, then reflecting a strong synergy between the repellents and PM. A similar trend was observed with the blood feeding rates, which were significantly lower for the mixtures than for each component alone.

**Conclusion:**

Synergistic interactions between organophosphates and repellents may be of great interest for vector control as they may contribute to increase the residual life of impregnated materials and improve the control of pyrethroid-resistance mosquitoes. These results prompt the need to evaluate the efficacy of repellent/non-pyrethroid insecticide mixtures against field populations of *An. gambiae *showing high level of resistance to Ops and pyrethroids.

## Background

Pyrethroid insecticides are currently the only chemicals recommended by World Health Organization Pesticide Scheme (WHOPES) for net impregnation because they show low mammalian toxicity and fast acting properties against mosquitoes [1]. Unfortunately, the knock-down resistance (*kdr*) gene conferring cross resistance to pyrethroids and DDT has become widespread in anopheline mosquitoes in Africa [2-5]. This resistance may represent a threat to the future success of malaria vector control programmes, based on insecticide-treated nets (ITNs) and indoor residual spraying (IRS). At present, there is uncertainty as to whether *kdr *undermines the effectiveness of ITN in areas of high prevalence. While experimental hut trial in Côte d'Ivoire [6] and Benin [6,7] demonstrated a survival advantage of mosquitoes being homozygous for the *kdr *resistance, other comparative trials between resistant and susceptible areas showed no apparent difference in the effectiveness of ITN [6,8,9]. The authors of this paper have previously suggested that resistant mosquitoes were less likely to be irritated by pyrethroid-treated nets than the susceptible and, therefore, alight for longer periods on ITNs and die [10,11]. This hypothesis was further explored by a randomized trial set up in Côte d'Ivoire which confirmed that ITNs remain effective in preventing malaria in areas where *Kdr *is prevalent [12].

Despite these controversial views, the reduced irritancy observed with ITNs against *kdr*-resistant mosquitoes represents a serious risk for personal protection. A typical example encountered in Benin was that significantly more individuals of the RS and RR genotypes blood fed in the presence of permethrin-treated nets than the susceptible SS [8]. Although carbamates and organophosphates are regarded as possible alternatives to pyrethroids [13,14], they may prove too hazardous for general use and may also select for insensitive acetylcholinesterase resistance in *An. gambiae *[13,15]. Developing alternative chemicals and/or vector control strategies to maintain an effective control of resistant mosquito populations has, therefore, become a priority.

In recent years, repellents have gained increasing interest in public health for protecting people against malaria vectors) [16-18]. DEET has been in use since the 1950s and is considered as the standard product against which all other repellents are measured [19]. Recently other active ingredients, known as IR3535 (ethyl butylacetylaminopropionate), KBR 3023 (Bayer), and PMD (para-menthane-3,8-diol) [19] have been formulated for skin application and showed equal or higher performances than DEET against mosquitoes [20]. Unfortunately, the issue with repellents concerns their short residual life which does not permit a long-term use in public health for personal protection. The application of repellents to fabrics, clothes or nets is a relatively unexplored topic which has potential benefits in terms of safety and cost as direct contact with the chemicals is reduced and persistence enhanced [21,22]. A recent experimental hut trial conducted in pyrethroid resistant area in Côte d'Ivoire, showed that standard lotions of DEET and IR3535 applied on nets showed similar performances than pyrethroid-treated nets during a 6 weeks period [23]. The observed residual effect of DEET on net is far higher than that observed for skin application (6–8 hours))[18] but shorter than that observed with standard ITNs.

Particularly promising is the good protection obtained from combined use of repellents on skin and ITN for personal protection in Pakistan [16,17]. This example of integrated vector control shows the gains that can be obtained if interventions are used jointly to cover for any limitation in individual interventions [24]. Another promising concept is to associate on nets a synthetic repellent with a non-pyrethroid insecticide to reconstitute pyrethroid features in terms of excito-repellency and knock-down effect. In a recent laboratory trial, a combination of propoxur (carbamate) and DEET on filter papers resulted in a synergistic effect which induced strong mortality and KD effect against susceptible and pyrethroid-resistant *Aedes aegypti *mosquitoes [25]. Such strategy may be promising for controlling malaria vectors which are becoming more and more resistant to the knock down and irritant effect of pyrethroids [8,11]. Through laboratory assays (tunnel test), the efficacy and persistence (mortality and blood feeding inhibition) of repellent-orgnanophosphate mixtures on polyester nettings against *An. gambiae*, the main malaria vector in Africa, were investigated.

## Methods

### Biological material

The reference susceptible strain of *An. gambiae *Kisumu was used. This strain, originating from Kenya, has been colonized for many years and is free from any detectable insecticide resistance mechanism.

### Insecticide and repellents

Three formulations, one organophosphate insecticide and two repellents, were evaluated on nets, separately or in mixture. Pirigrain^® ^250 is an Emulsifiable Concentrate formulation (EC) containing 25% pyrimiphos methyl (PM) and manufactured by Compagnie Générale des Insecticides (CGI, France). KBR 3023 (hydroxyethyl isobuthyl piperidine carboxyilate) is formulated as a liquid concentrate containing 25% of active ingredient. DEET (diethyl-3-methylbenzamide) is also formulated as a liquid concentrate containing 30% of active ingredient. The two experimental formulations of repellents are designed for clothing application and developed by Osler Company (France).

### Net treatment

Netting samples to be tested in the tunnel apparatus were 75 denier multi-filament polyester, mesh 156, provided by Paluteck^®^, Benin. They were treated alone or in combination at 10 g/m^2 ^with DEET and KBR 3023 and 150 mg/m^2 ^with PM . These dosages have been selected after preliminary tests, as the lower dosages inducing 100% mortality in tunnel. Because repellents are volatile compounds, tests under tunnels were carried out near after the impregnation process (6 hours). Then blood-feeding inhibition and mortality were evaluated twice a week until efficacy dropped to values below 30%.

### Study design and statistical analysis

The tunnel system is composed of a square glass cylinder, 25 cm high, 21 cm wide, 60 cm long, with a square of netting sizing 25 × 25 cm with nine 1 cm diameter holes fixed into a frame which slots across the tunnel dividing it into two chambers. In the bait chamber, a guinea pig is housed unconstrained in a cage and provided with food and water, and in the other chamber, 100 unfed female mosquitoes aged 5–8 days are released at dusk and left overnight in the dark. The following morning, the number of mosquitoes found live or dead, fed or unfed in each compartment was recorded.

Blood feeding reduction was assessed by comparing the proportion of blood-fed females (whether they were alive or dead) in treated and control tunnels. With each treatment, the blood feeding Inhibition rate (BFI) was calculated using the following formula:

BFI=100−(Treated∗100)Control     Eq.1
 MathType@MTEF@5@5@+=feaafiart1ev1aaatCvAUfKttLearuWrP9MDH5MBPbIqV92AaeXatLxBI9gBaebbnrfifHhDYfgasaacH8akY=wiFfYdH8Gipec8Eeeu0xXdbba9frFj0=OqFfea0dXdd9vqai=hGuQ8kuc9pgc9s8qqaq=dirpe0xb9q8qiLsFr0=vr0=vr0dc8meaabaqaciaacaGaaeqabaqabeGadaaakeaacqWGcbGqcqWGgbGrcqWGjbqscqGH9aqpcqaIXaqmcqaIWaamcqaIWaamcqGHsisldaWcaaqaaiabcIcaOiabdsfaujabdkhaYjabdwgaLjabdggaHjabdsha0jabdwgaLjabdsgaKjabgEHiQiabigdaXiabicdaWiabicdaWiabcMcaPaqaaiabdoeadjabd+gaVjabd6gaUjabdsha0jabdkhaYjabd+gaVjabdYgaSbaacaWLjaGaaCzcaiabbweafjabbghaXjabb6caUiabigdaXaaa@5288@

Overall mortality was measured by pooling both immediate (12 hrs) and delayed (24 hrs) mortality of mosquitoes from the two sections of the tunnel. When control mortality exceeded 5%, treatment-induced mortality rates were corrected using the Abbott formula [29]:

CorrectedMortality=(Treated−Control)(100−Control)     Eq.2
 MathType@MTEF@5@5@+=feaafiart1ev1aaatCvAUfKttLearuWrP9MDH5MBPbIqV92AaeXatLxBI9gBaebbnrfifHhDYfgasaacH8akY=wiFfYdH8Gipec8Eeeu0xXdbba9frFj0=OqFfea0dXdd9vqai=hGuQ8kuc9pgc9s8qqaq=dirpe0xb9q8qiLsFr0=vr0=vr0dc8meaabaqaciaacaGaaeqabaqabeGadaaakeaacqWGdbWqcqWGVbWBcqWGYbGCcqWGYbGCcqWGLbqzcqWGJbWycqWG0baDcqWGLbqzcqWGKbazcqWGnbqtcqWGVbWBcqWGYbGCcqWG0baDcqWGHbqycqWGSbaBcqWGPbqAcqWG0baDcqWG5bqEcqGH9aqpdaWcaaqaaiabcIcaOiabdsfaujabdkhaYjabdwgaLjabdggaHjabdsha0jabdwgaLjabdsgaKjabgkHiTiabdoeadjabd+gaVjabd6gaUjabdsha0jabdkhaYjabd+gaVjabdYgaSjabcMcaPaqaaiabcIcaOiabigdaXiabicdaWiabicdaWiabgkHiTiabdoeadjabd+gaVjabd6gaUjabdsha0jabdkhaYjabd+gaVjabdYgaSjabcMcaPaaacaWLjaGaaCzcaiabbweafjabbghaXjabb6caUiabikdaYaaa@7014@

The Lethal Time (LT) and Biting Inhibition Time (BIT) were afforded by each treatment by fitting a sigmoidal time-response model with GOSA^® ^software [26] using the following formula:

Y=min⁡+(max⁡−min⁡)1+10(log⁡LT95−log⁡.x)∗slope     Eq.3
 MathType@MTEF@5@5@+=feaafiart1ev1aaatCvAUfKttLearuWrP9MDH5MBPbIqV92AaeXatLxBI9gBaebbnrfifHhDYfgasaacH8akY=wiFfYdH8Gipec8Eeeu0xXdbba9frFj0=OqFfea0dXdd9vqai=hGuQ8kuc9pgc9s8qqaq=dirpe0xb9q8qiLsFr0=vr0=vr0dc8meaabaqaciaacaGaaeqabaqabeGadaaakeaacqWGzbqwcqGH9aqpcyGGTbqBcqGGPbqAcqGGUbGBcqGHRaWkdaWcaaqaaiabcIcaOiGbc2gaTjabcggaHjabcIha4jabgkHiTiGbc2gaTjabcMgaPjabc6gaUjabcMcaPaqaaiabigdaXiabgUcaRiabigdaXiabicdaWmaaCaaaleqabaGaeiikaGIagiiBaWMaei4Ba8Maei4zaCMaemitaWKaemivaq1aaSbaaWqaaiabiMda5iabiwda1aqabaWccqGHsislcyGGSbaBcqGGVbWBcqGGNbWzcqGGUaGlcqWG4baEcqGGPaqkcqGHxiIkcqWGZbWCcqWGSbaBcqWGVbWBcqWGWbaCcqWGLbqzaaaaaOGaaCzcaiaaxMaacqqGfbqrcqqGXbqCcqqGUaGlcqaIZaWmaaa@6200@

where (*x*) is the time (in days) entered without any transformation (i.e. not in logarithmic form). *Y *is the response (LT or BIT) which varies between a minimum (min) and a maximum (max). *LT*_95 _and *BIT*_95 _are respectively the Lethal Time and Biting Inhibition Time (days) for *Y *95% mortality or blood feeding between min and max respectively, i.e. the time after which 95% mosquitoes are dead or still unfed. *Slope *represents the slope of the curve at its midpoint.

In order to detect any synergy between PM and DEET or KBR, the results observed with the two mixtures with those theoretically expected in the absence of any interaction (uncorrelated joint action) between the two compounds were compared [27]. The expected mortality was calculated by multiplying the survival rates of each compound tested separately at each time class and subtracting the result from 100%, as follow:

*Exp *= 1 -((1 - *mort*^*rep*^)*(1-*mort*^*pm*^))     Eq.4

Expected values of mortality and blood feeding inhibition rate were also fitted using the same sigmoidal time-response model. Then observed and expected LT_95 _and BIT_95 _were compared. There was synergy when the observed results were significantly higher than the expected one. Conversely, there was antagonism when the observed results were significantly lower than the expected one. The differences between two LT_50 _and two BIT_50 _values were considered as significant if their 95% confidence intervals (CI_95_) did not overlap.

## Results

**Figure 1:**
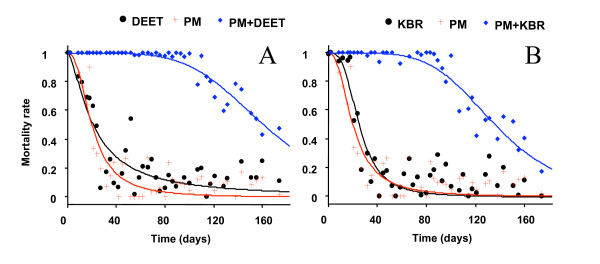
**Decline with time in treated nets efficacy**. Mortality of *An. gambiae *Kisumu during overnight exposure to treated netting in tunnel test apparatus; Pyrimiphosmethyl was used at 150 mg/m^2^alone and combined with **(a) **DEET 10 g/m^2 ^and **(b) **KBR 10 g/m^2^. Surimposed curves drawn according to sigmoidal time – response model of equation (3) whose parameters are shown in Table 1.

**Figure 2:**
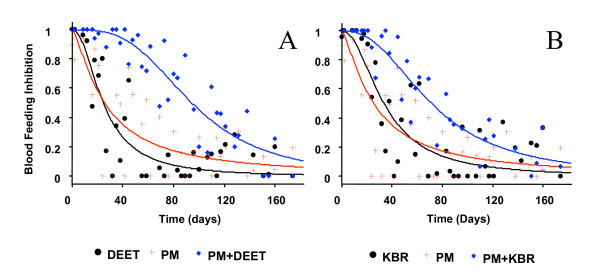
**Decline with time in blood feeding inhibition**. Blood feeding Inhibition provided by treated netting against *An. gambiae *Kisumu during overnight tests in tunnel test apparatus; Pyrimiphosmethyl was used at 150 mg/m^2^alone and combined with **(a) **DEET 10 g/m^2 ^and **(b) **KBR 10 g/m^2^. Surimposed curves drawn according to sigmoidal time – response model of equation (3) whose parameters are shown in Table 1.

The mortality and blood feeding inhibition rates recorded during the evaluation are shown in Figure 1 and 2. Statistics are summarized in Table.

### Lethal effect

When freshly treated, mortality of *An. gambiae *was 100% with each type of treatment (singles and mixtures). There was a more rapid decline in activity over time of the mono treatments than the mixtures. At their respective dosage, the LT_95 _of each chemical never exceeded 10 days (LT_95_^PM ^= 5.46 days ± 2.14; LT_95_^DEET ^= 2.79 days ± 1.68; LT_95_^KBR ^= 9.43 days ± 3.17). KBR showed a LT_95_similar to PM but significantly longer than DEET. The two mixtures DEET/PM and KBR/PM (Figure 1a and 1b) killed 95% mosquitoes for more than 60 days (LT_95_^PM+DEET^= 87 ± 11; LT_95 _^PM+KBR ^= 73 ± 9).

### Blood feeding inhibition

Onset inhibition of blood feeding was total (100%) with every treatment. The time required to inhibit 95% of the blood feeding (BIT_95_) was three days (± 3), six days (± 3) and eight days (± 5) for PM, DEET and KBR, respectively. There was no significant difference between the three molecules tested (Table 1). PM+DEET mixture induced 95% blood feeding inhibition for more than one month (BIT_95_^PM+DEET ^= 37 ± 10 days), whereas the effect lasted for three weeks only for the PM+KBR mixtures (BIT_95 _^PM+KBR ^= 21 ± 8 days). The BIT of the mixtures was significantly longer than those observed when each compound was used separately (Figure 2a and 2b).

**Table 1:** Summary statistics for nets treated with Pyrimiphos-methyl (PM 150 mg/m^2^), DEET and KBR (both at 10 g/m^2^), alone and in combination against susceptible *An. gambiae*. Slope (95% CI), Lethal Time for 50 and 95% (LT_50–95 _in days), Biting Inhibition Time 50 and 95% (BIT_50–95 _in days).

	Mortality						Blood Feeding Inhibition					
		
Insecticide/repellent	slope	(95%CI)	LT_50_	(95%CI)	LT_95_	(95%CI)	slope	(95%CI)	BIT_50_	(95%CI)	BIT_95_	(95%CI)
PM	-2.29^ab^	± 0.64	19.67^a^	± 2.97	5.46^ab^	± 2.14	-1.33^a^	± 0.59	25.46^a^	± 9.33	2.80^a^	± 2.93
DEET	-1.46^a^	± 0.40	20.91^ac^	± 4.75	2.79^a^	± 1.68	-2.04^a^	± 0.78	24.38^a^	± 5.27	5.79^a^	± 3.44
KBR	-3.04^b^	± 0.98	24.79^ac^	± 3.19	9.43^b^	± 3.17	-2.06^a^	± 0.81	33.51^a^	± 7.39	8.03^a^	± 4.84
PM+DEET	-4.81^c^	± 0.92	161.03^b^	± 6.63	87.32^c^	± 10.84	-3.18^a^	± 0.90	94.34^b^	± 7.99	37.44^b^	± 10.29
PM+DEET expected	-1.37^a^	± 0.44	31.14^c^	± 7.56	3.65^ab^	± 2.66	-1.57^b^	± 0.60	52.46^c^	± 12.04	8.09^a^	± 6.11
PM+KBR	-4.79^c^	± 0.96	135.39^b^	± 5.22	73.30^c^	± 9.48	-2.40^a^	± 0.67	71.42^c^	± 8.10	21.04^bc^	± 7.54
PM+KBR expected	-1.70^ab^	± 0.54	30.45^c^	± 6.57	5.41^ab^	± 3.19	-1.74^b^	± 0.59	58.09^c^	± 10.72	10.79^ac^	± 6.45

Numbers in the same line sharing the same superscript letter do not differ significantly (Confidence Intervals are overlapping)

### Synergy

There were highly significant differences between expected and observed LT_95 _of PM+DEET and PM+KBR (95% CI do not overlap) indicating a strong synergy between PM and the repellents tested in terms of mortality (Table 1). A strong synergy was also noted with blood feeding inhibition, the observed BIT_95 _of the PM+DEET mixture being significantly greater than expected (Table 1). For the mixture PM+KBR, however, there was no significant difference between expected and observed blood feeding inhibition (overlapping of the confidence intervals), then suggesting a simple additive effect for blood feeding inhibition.

## Discussion

When used separately, DEET and KBR on nets both induced, even for a few days, high mortality rates (more than 95%). This is a confirmation that DEET is not only a behavioural modifying chemical but also a toxicant as previously demonstrated by several authors [23,28-30]. The molecular events involved in DEET toxicity in insects is currently under investigation [31,32]. More surprising was the mortality observed with KBR 3023 in tunnel apparatus. Indeed, KBR 3023 did not show insecticidal properties as DEET in previous works [30] but it was not tested on impregnated materials. However, the dose of KBR used in previous works (2 g/m^2 ^on filter papers) as well as the time of exposure (1 h in WHO test kits) was far below the one used in this study (10 g/m^2 ^and 12 hrs exposure). As far as residual efficacy is concerned, results with DEET are similar to those obtained in tunnel tests by N'Guessan et al. [23] in term of mortality but strongly different in term of blood feeding inhibition (three weeks of total protection vs. four days in this study). This difference may be due to the mosquito species used instead (*Culex quinquefasciatus*) and/or the formulations (DEET in alcohol versus liquid concentrate).

When used in mixtures, results clearly indicate that mixing an OP with a repellent significantly improve, at least under tunnel, the efficacy of nets against anopheline mosquitoes, both in terms of mortality and blood feeding inhibition. This is a confirmation of a previous study that showed a strong synergistic interaction between DEET and propoxur (carbamate) against *A. aegypti *mosquitoes [25]. In this study, thanks to synergy, it appears that a mixture combining an organophosphate insecticide with a repellent (DEET or KBR3023), is as effective as a most pyrethroids recommended by WHO for the treatment of mosquito nets [33]. Indeed, the overall efficacy of the mixtures was maintained for more than four months with PM+KBR3023 and five months PM+DEET in tunnel tests. Moreover, the tested mixture showed a residual effect longer than the one observed by other authors with chlorpyriphos-methyl (one month at 100 mg/m^2^) (N'Guessan personal communication) and approximately similar to the one observed with PM alone (7 months versus 5 in our study) [34]. However, the dosage used was very high (1,000 mg/m^2^) compared to 150 mg/m^2 ^in the present study.

The great efficacy of these two repellent/OPs mixtures may offer interesting prospects for controlling malaria vectors. It could be a promising strategy to manage *kdr*-resistant mosquitoes [25], since the non-pyrethroid mixture on net seems to be as effective as a pyrethroid insecticide. Another advantage is the considerable reduction of the insecticide amount on net, therefore, pledging the use of OPs on net. Next steps will consist in evaluating the efficacy and residual activity of these mixtures in experimental huts in the field. It would be also interesting to investigate the effectiveness of such mixtures against mosquitoes bearing other resistance mechanisms, such as the insensitive acetylcholine esterase (AChE^*1R*^)[35].

## Competing interests

The author(s) declare that they have no competing interests.

## Authors' contributions

CP carried out the laboratory evaluation, analyzed and interpreted data and drafted the manuscript. VC substantially helped draft the manuscript. PB and AO helped to carry out the laboratory evaluation. RN helped draft the manuscript. BL participated to design the study. JMH designed the study, interpreted the data and helped draft the manuscript.
